# Exploratory Analysis of Image-Guided Ionizing Radiation Delivery to Induce Long-Term Iron Accumulation and Ferritin Expression in a Lung Injury Model: Preliminary Results

**DOI:** 10.3390/bioengineering11020182

**Published:** 2024-02-14

**Authors:** Amira Zaher, Bryce Duchman, Marina Ivanovic, Douglas R. Spitz, Muhammad Furqan, Bryan G. Allen, Michael S. Petronek

**Affiliations:** 1 Department of Radiation Oncology, Division of Free Radical and Radiation Biology, University of Iowa, Iowa City, IA 52242, USA; amira-zaher@uiowa.edu (A.Z.); douglas-spitz@uiowa.edu (D.R.S.); 2 Division of Pulmonary, Critical Care, Sleep Medicine & Physiology, UC San Diego Health, San Diego, CA 92093, USA; brduchman@health.ucsd.edu; 3 Department of Pathology and Laboratory Medicine, Loyola University Health System, Loyola University, Chicago, IL 60660, USA; marina-ivanovic@uiowa.edu; 4 Department of Internal Medicine Division of Hematology and Oncology, University of Iowa, Iowa City, IA 52242, USA; muhammad-furqan@uiowa.edu

## Abstract

Background: Radiation therapy (RT) is an integral and commonly used therapeutic modality for primary lung cancer. However, radiation-induced lung injury (RILI) limits the irradiation dose used in the lung and is a significant source of morbidity. Disruptions in iron metabolism have been linked to radiation injury, but the underlying mechanisms remain unclear. Purpose: To utilize a targeted radiation delivery approach to induce RILI for the development of a model system to study the role of radiation-induced iron accumulation in RILI. Methods: This study utilizes a Small Animal Radiation Research Platform (SARRP) to target the right lung with a 20 Gy dose while minimizing the dose delivered to the left lung and adjacent heart. Long-term pulmonary function was performed using RespiRate-x64image analysis. Normal-appearing lung volumes were calculated using a cone beam CT (CBCT) image thresholding approach in 3D Slicer software. Quantification of iron accumulation was performed spectrophotometrically using a ferrozine-based assay as well as histologically using Prussian blue and via Western blotting for ferritin heavy chain expression. Results: Mild fibrosis was seen histologically in the irradiated lung using hematoxylin and eosin-stained fixed tissue at 9 months, as well as using a scoring system from CBCT images, the Szapiel scoring system, and the highest fibrotic area metric. In contrast, no changes in breathing rate were observed, and median survival was not achieved up to 36 weeks following irradiation, consistent with mild lung fibrosis when only one lung was targeted. Our study provided preliminary evidence on increased iron content and ferritin heavy chain expression in the irradiated lung, thus warranting further investigation. Conclusions: A targeted lung irradiation model may be a useful approach for studying the long-term pathological effects associated with iron accumulation and RILI following ionizing radiation.

## 1. Introduction

Radiation-induced lung injury (RILI) is a common clinical challenge lung cancer patients experience following radiation therapy (RT). Nearly 77% of lung cancer patients have an evidence-based indication for RT as part of their therapeutic regimen, particularly in patients with stage I–II non-small-cell lung cancer (NSCLC) who are medically inoperable or decline surgery, stage III NSCLC, or limited stage small-cell lung cancer (SCLC) [1]. However, lung tissues are sensitive to RT and may give rise to RILI. Chronic RILI may lead to the onset of pulmonary fibrosis in up to 20% of RT-treated lung cancer patients. Clinical thoracic radiation doses range between 30 and 60 Gy and depend upon the stage of disease, plan for surgical intervention, and proximity of dose-limiting organs [2,3]. The incidence of RILI is often the result of normal lung tissue receiving ≥20 Gy of radiation during treatment [4,5]. RILI may be further exacerbated by increased dose per fraction, such as with stereotactic body radiation therapy (SBRT) [6].

RILI may result in dyspnea, significantly diminishing a patient’s quality of life; thus, there is a pressing need to reduce the incidence and severity of RILI [7]. There is evidence that localized iron overload is a pathologic response to RT and is thought to contribute to the development of tissue fibrosis in multiple organs, including the lung [8]. Thus, iron metabolism is an emerging target for the mitigation of RILI. Iron accumulation can induce ferroptosis, inflammation, and mitochondrial dysfunction. The use of ferroptosis inhibitors and iron chelation therapy can mitigate the long-term effects of RT [9,10,11,12,13]. One of the key iron homeostasis proteins is ferritin heavy chain (FtH), the catalytic subunit of ferritin known to respond to iron overload through its ferroxidase activity converting Fe^2+^ to Fe^3+^ that is then stored in the light chain subunit of ferritin [14,15,16,17]. Circulating ferritin levels are elevated under conditions of severe lung injury and inflammation, correlating with decreased lung function [18,19,20,21]. While most of the work regarding the relationship of iron to interstitial lung injury concerning ferritin has remained largely focused on elevated circulating ferritin as a prognosticator of disease progression, the intracellular role of ferritin remains elusive. However, it can be reasonably hypothesized that the elevated serum ferritin levels associated with lung injury may be related to an increase in intracellular ferritin expression in the damaged organ. Thus, the goal of this study is to evaluate the potential utility of a targeted, single-lung irradiation approach to model radiation-induced fibrosis and study iron accumulation and ferritin heavy chain immunoreactive protein in animals.

## 2. Materials and Methods

### 2.1. Animals

Six-week-old C57BL6/J (Jackson Laboratories) male mice were utilized for this study due to the increased radiosensitivity of the strain and its susceptibility to pulmonary fibrosis [22,23,24,25]. A total of 10 animals (5 animals per group) were housed at the University of Iowa animal facility in a temperature-controlled room with 12 h light/12 h dark cycles. For the duration of the study (36 weeks), animals were monitored 3–4 times weekly for signs of distress or disease. Criteria for euthanasia (humane end point) were defined as weight loss >30%. Two animals met the end point requirements in this study and were euthanized immediately. Euthanasia was accomplished using 100% CO_2_ gas administration for 1 min until breathing stopped. Confirmation of euthanasia was accomplished using cervical dislocation and harvesting of the lungs. The remaining 8 animals did not meet the humane end point requirements but were euthanized at end of the study for tissue collection purposes. All procedures were approved by the University of Iowa IACUC (Protocol #2121022, 2 February 2023).

### 2.2. Radiation Treatment Design

**Figure 1:**
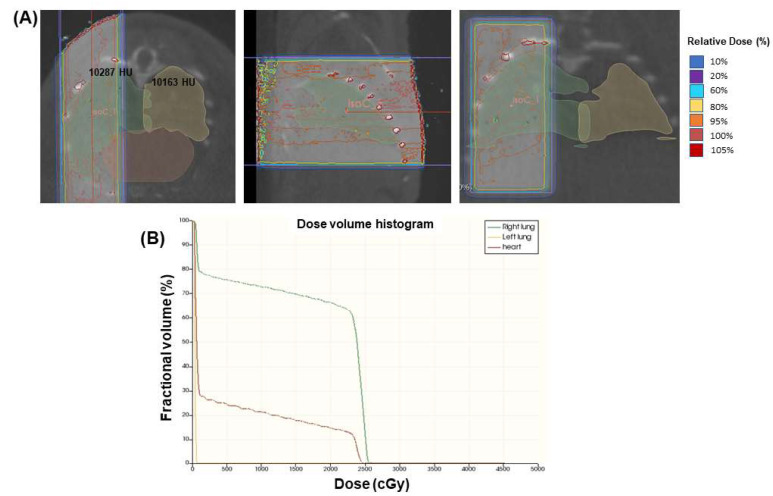
Single-lung-targeted radiation treatment design and validation. To spare surrounding tissues (left lung and heart) while maintaining a high dose to the right lung, 25 Gy was prescribed to a single isocenter within the right lung (green), with the left lung (yellow) and heart (brown) being considered OARs for sparing (**A**). Each animal was treated individually and validated by generating a DVH to confirm adequate dosing (**B**).

Mice were treated under isoflurane anesthesia with a single fraction of x-ray irradiation targeting the right lung using an Xstrahl Small Animal Radiation Research Platform (SARRP). The target dose to the right lung was 20 Gy total, as RILI is known to result from normal lung doses ≥ 20 Gy [6]. To achieve this target dosage, a single isocenter in the right lung was prescribed 25 Gy (Figure 1A). Treatment plans for every animal were generated using the MuriPlan.3.0.0 software. Single-lung targeting was performed using the multivariable collimator with a field size of 15 × 7 mm to avoid organs at risk (OAR), defined as the left lung, which was used as an internal control, and the heart to mitigate potential concurrent cardiotoxicities that could confound the results. The target volume (right lung) and OARs (left lung and heart) were individually defined on the pretreatment cone-beam CT (CBCT) image, and an individual treatment plan was generated for every animal. No differences in lung density were observed between the right and left lungs. A dose volume histogram (DVH) was generated to ensure appropriate dosing to the target volume and OAR sparing (Figure 1B). The mean dose to the right lung was 17.2 ± 1.4 Gy with a maximum dose of 44.5 ± 6.8 Gy. The mean dose to the left lung was 3.1 ± 1.2 Gy with a maximum dose of 16.7 ± 8.7 Gy. The mean dose to the heart was 3.1 ± 1.5 Gy with a maximum dose of 27.8 ± 5.9 Gy. The large maximum dose to the heart typically occurs in the medial region that largely overlaps with the right lung, but the overall dose to the entire organ was able to be maintained near 0 Gy in those regions largely overlapping with the left lung. Based on the DVH measurements, <20% of the heart and <2% of the left lung received any dose. The radiation treatment was delivered using a two-beam AP/PA approach (0°/179°), with both beams having equal weighting (50%).

### 2.3. Threshold Analysis of CBCT Images

Using the 3D Slicer software 5.2.2, a threshold for normal-appearing lung volume (NALV) was obtained and used to quantify NALV in cone beam CT (CBCT) images (220 µm resolution) acquired on the Xstrahl SARRP 9 months after radiation and prior to euthanasia. Following the application of a threshold (787.11–6462.37 HU generated using a normal, healthy lung) to remove non-NALV, NALV (mm^3^) was calculated using the segmentation statistics package in 3D Slicer. Three-dimensional models were generated within 3D Slicer.

### 2.4. Breathing Rate Testing

Mouse respiratory rates were measured at baseline (prior to irradiation or sham) and every 4 weeks after irradiation or sham until death or euthanasia. A clear isoflurane sedation box connected to the Vetamec Compact II small animal anesthetic machine was utilized for sedation. In groups of 3–5 mice, mice were placed in the isoflurane sedation box with 3 L per minute (LPM) supplemental oxygen (no isoflurane). Isoflurane at a 2% concentration was then initiated, and mice were induced for 210 s. At 210 s, the isoflurane sedation box lid was opened for exactly 30 s to allow sedated mice to be oriented in the same direction and without overlapping body parts. The box lid was closed, and isoflurane 2% and 3 LPM supplemental oxygen were continued. A Nexigo N980P (T/N 920PJH; P/N USBL-625JH) wideview camera was placed on top of the clear isoflurane sedation box. A video recording of each mice group was started exactly 300 s after isoflurane initiation, and mice were recorded for 310 s (total of 610 s of isoflurane 2% sedation by the end of video recording). Between groups, the isoflurane box was allowed to “wash out” with 3 LPM oxygen (without isoflurane) with the lid open for 120 s plus with the lid closed for an additional 120 s. The process was repeated until all mice were sedated and video recorded. As previously described and validated, the open-source software RespiRate-x64 was utilized to track respiratory motion in video recordings and to calculate the respiratory rate for each mouse [23]. Video recordings were analyzed in 30 s segments at specific timepoints. In our validation measurements, the calculated respiratory rate matched the visually measured respiratory rate to within 5 breaths per minute (bpm) in all cases (and typically < 2 bpm) if the software’s respiratory motion waveform standard deviation (SD) was <0.1800. Thus, if the recorded respiratory motion waveform SD was not less than 0.1800, then video recordings were visually reviewed, and respiratory rate was visually measured instead.

### 2.5. Tissue Staining

Immediately following euthanasia at 9 months, irradiated and unirradiated lungs were harvested and fixed in 10% neutral buffered formalin solution. Following fixation, tissues were processed and paraffin-embedded by the University of Iowa Comparative Pathology Laboratory. Embedded tissues were sectioned and mounted on glass histology slides and stained with H&E, trichrome, and Prussian blue at the University of Iowa Comparative Pathology Laboratory. Staining was evaluated and scored by a blinded pulmonary pathologist. Szapiel score was used to assess pulmonary fibrosis on a score from 0 to 3, where 0 indicates no fibrosis, 1 indicates mild localized fibrosis with minimal alveolar thickening, 2 indicates moderate fibrosis in >20 and <50% of the lung with fibrous bands or masses present, and 3 indicates severe fibrosis in ≥50% of the lung [26].

### 2.6. Colorimetric Quantification of Iron

Freely cheatable iron concentrations in lung tissue were determined using a ferrozine-based colorimetric assay previously described by Abbasi et al. [27] with minor adaptations. Flash-frozen lungs were homogenized in 1× RIPA lysis buffer (Sigma-Aldrich, Burlington, MA, USA) and sonicated. Following incubation on ice for 5 min, lysates were centrifuged at maximum speed for 10 min. Supernatants were collected and moved to fresh microcentrifuge tubes. In total, 120–150 µL of the supernatant was added to 100 µL of assay buffer (5 mM ferrozine, 1.25 M ammonium acetate, and 10 mM ascorbate). Reaction mixtures were then transferred to a clear 96-well plate, and the Fe^2+^ concentration was determined spectrophotometrically by detecting the formation of Fe^2+^–ferrozine complex at 562 nm. [Fe] was calculated using Beer’s Law:

A562(A.U.) = ε_562_ ∗ [Fe] ∗ L

where A562 is absorbance at 562 nm, ε_562_ is the molar extinction coefficient for a Fe^2+^–ferrozine complex = 27,900 M^−1^ cm^−1^, [Fe] is the calculated Fe concentration (M), and L is the pathlength for 200 µL of liquid ≈ 0.55 cm. [Fe] values were normalized to protein concentrations calculated using the DC™ protein assay kit (Bio-Rad, Hercules, CA, USA).

### 2.7. Western Blotting

In total, 25 μg of total protein from lung lysates was used for Western blotting. Samples were run at 115 V on a 4–20% precast gradient gel (Bio-Rad) for 60 min. Proteins were transferred to a PVDF membrane at 4 °C, 100 V, for 60 min. Following transfer, the membrane was blocked using 5% nonfat dry milk in 0.2% TBS-Tween (TBST) for 90 min at room temperature. The membrane was incubated overnight at 4 °C with the following primary antibodies: ferritin heavy chain (1:2000, Abcam, Waltham, MA, USA), and GAPDH was used as a loading control (1:1000; Santa Cruz). Following 3 × 10 min washes with TBST, the membrane was incubated with horseradish peroxidase-conjugated goat antimouse secondary antibody (1:10,000–1:20,000; cell signaling, Danvers, MA, USA) for 1 h at room temperature. Following 3 × 10 TBST washes, Super Signal West Pico Chemiluminescent Substrate (Thermo Scientific, Waltham, MA, USA) was added to the membrane and exposed on an X-ray film (Research Products International, Mount Prospect, IL, USA).

### 2.8. Statistical Analysis

Student’s *t* test, the Wilcoxon test, and one-way ANOVA or two-way ANOVA with Tukey’s post hoc test were used to test for differences between groups accordingly. GraphPad Prism 8.0 (GraphPad Software Inc., La Jolla, CA, USA) was used for statistical analysis. *p* < 0.05 was considered statistically significant.

## 3. Results

### 3.1. Survival Outcomes

**Figure 2:**
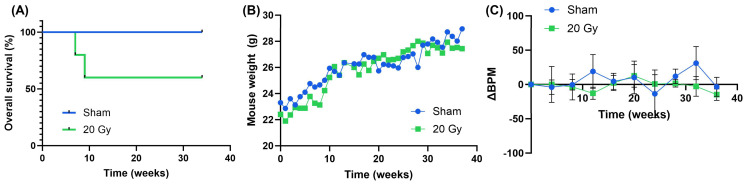
Survival and pulmonary function outcomes. (**A**) Overall survival in irradiated animals showing a 40% decrease in the irradiated animals compared to sham controls. (**B**) Daily body weights (g) in sham controls and irradiated mice showing no changes over time. (**C**) Changes in breathing rates (BPM) for controls and irradiated animals over time; no changes were detected between the two groups. Data are represented as means, with error bars representing the standard error of the mean (SEM).

The overall outcomes of our targeted treatment strategy were characterized. As anticipated, there were no deaths that occurred in the control mice receiving sham radiotherapy. In contrast, by study duration (36 weeks), 40% of the irradiated cohort had required euthanasia (*p* = 0.136) (Figure 2A). There were no differences in weight between the sham and irradiated groups throughout the study duration, indicating no significant systemic distress (*p* = 0.4237) (Figure 2B). There was also no difference in breathing rates throughout the study between the groups (*p* = 0.1503) (Figure 2C). Thus, irradiation of the sub-total organ volume allows for obtaining a sustained pulmonary capacity.

To contextualize these results, our overall survival results were compared to other reports using whole thorax irradiation (WTI) (Table 1). This study uses a prescribed dose of 25 Gy to a single lung (right lung) to achieve a target dose of 20 Gy, in contrast to other studies which used a prescribed dose of 20 Gy in WTI. We were unable to reach a median overall survival within 36 weeks, while the typical median overall survival that is reported for 20 Gy WTI in female C57BL6/J mice has a range of 13.4–22.3 weeks [28,29,30,31,32,33,34]. Thus, our targeted, single-lung approach may provide extended survival relative to WTI, allowing for a more robust study of long-term radiation-induced lung injury.

### 3.2. Long-Term Radiation-Induced Lung Injury Was Confirmed in the Single-Lung Irradiation Model

**Figure 3:**
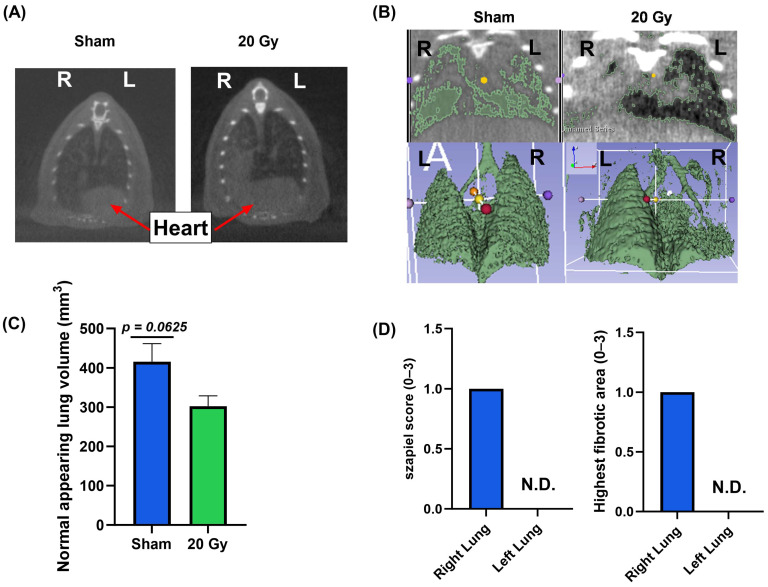
Confirmation of long-term radiation-induced lung injury at 9 months following exposure. (**A**) The left image shows a representative scan of a sham control without significant consolidation in either lung. The right image shows a representative scan of an irradiated lung exhibiting consolidation in the right lung with minimal effect to the left lung. (**B**) Representative CBCT images with NALVs delineated (top) and associated 3D models (bottom) of sham and irradiated lungs showing normal-appearing lung volume (NALV) changes with radiation. Three-dimensional reconstructions were rotated 180° to better visualize the anatomy. (**C**) Quantification of NALV in sham and irradiated lungs. (**D**) Szapiel score (0–3 scale) and highest fibrotic score (0–3 scale) for the left and right lungs of irradiated animals (*n* = 3), indicating injury was present only in the right lungs. Error bars represent standard error of the mean (SEM).

Prior to euthanasia, the extent of tissue damage was evaluated using CBCT imaging. Qualitative analysis of the CBCT images revealed evidence of opacities consistent with lung injury (Figure 3A). CBCT images were quantitatively analyzed to further assess potential radiographic evidence of injury using an automated image threshold approach in Slicer 3D software to generate the NALVs (Figure 3B). Three-dimensional reconstruction of the NALV reveals a loss of anatomical structure in the irradiated lung. Moreover, there was a decrease in NALV trending towards statistical significance in irradiated mice as compared to sham controls (302.6 ± 26.2 versus 416.0 ± 45.6 mm^3^, *p* = 0.0625 Figure 3C). Following euthanasia, lung tissue harvested from the irradiated mice was stained with H&E and trichrome to characterize long-term pathology, with the unirradiated (left) lungs being used as an internal control. To characterize the onset of fibrosis, the Szapiel score was used [26], where the irradiated right lung showed mild fibrotic onset (Szapiel score = 1), which was not apparent in the left lung (Szapiel score = 0) (Figure 3D). Similarly, the highest fibrotic score revealed the same trend (Figure 3D). Thus, it appears that we were able to induce pathologic radiation-induced changes in the irradiated right lung while sparing the left lung, allowing it to serve as an internal control.

### 3.3. Increased Iron Content and Ferritin Heavy Chain Levels in Irradiated Lung Tissue

**Figure 4:**
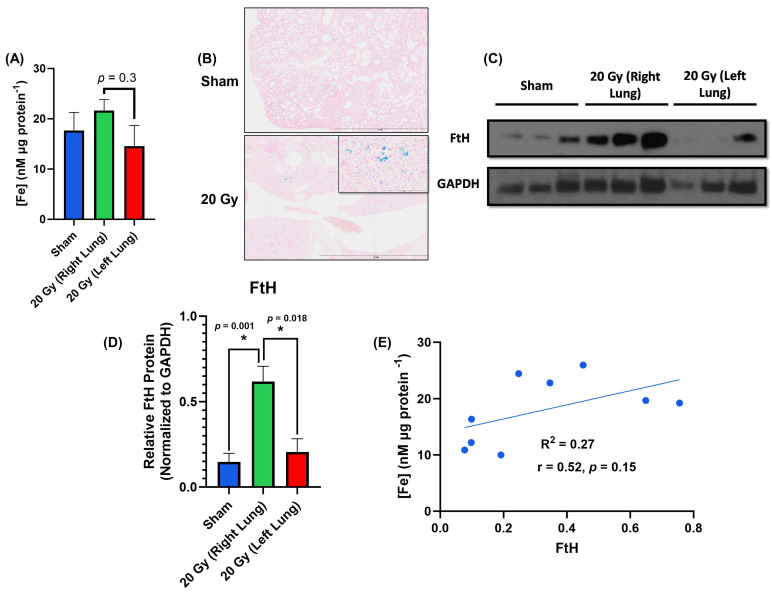
Changes in iron content and FtH levels in sham, irradiated, and spared lungs. (**A**) Iron content (per µg of protein) in the sham control lungs, right lungs (25 Gy), and matched left lungs, *n* = 3 per group. Iron content increased in the right lungs and decreased in the matched spared left lungs (*p* = 0.3). (**B**) Compared to sham control, representative images of Prussian blue staining showed positive staining in the irradiated right lung (needs quantitation). (**C**) Western blot results showing FtH in the irradiated right lung compared to sham controls and matched left lungs. (**D**) Relative FtH normalized to GAPDH using Western blotting showing a significant increase in FtH in the right lung compared to sham controls and matched left lungs. (**E**) Correlation between iron content detected using the ferrozine assay and FtH levels detected using Western blotting showing a trend towards a statistically significant positive correlation. * *p* < 0.05 considered statistically significant.

Following model validation, iron accumulation was evaluated. Using a ferrozine-based detection method, an increase in iron content was observed in the irradiated lungs compared to both sham and internal unirradiated controls (mean = 17.68, 21.64, and 14.57 Fe µg protein ^−1^, respectively, Figure 4A). Due to limited sample availability (*n* = 3 per treatment), differences in [Fe] were not statistically significant between sham controls and right lungs (*p* = 0.7) or spared left lungs and right lungs (*p* = 0.3); however, this trend was consistent with previous studies in the literature showing increased iron content following irradiation [11,35,36,37]. Furthermore, we confirmed these findings on iron accumulation via Prussian blue staining, where only irradiated lungs showed evidence of Prussian-blue-positive cells (Figure 4B). Moreover, FtH levels in the right lung were significantly higher compared to sham controls and the spared left lungs (*p* = 0.001, *p* = 0.018, respectively) (Figure 4C,D), consistent with the induction of FtH in response to increased tissue iron content. Our data showed a trend of positive correlation (R^2^ = 0.27, r = 0.5, *p* = 0.15) between [Fe] and FtH protein levels (Figure 4E). Although not statistically significant (*p* = 0.15), this finding is consistent with previous studies showing the relationship between FtH and [Fe] [38,39]. Therefore, it appears that ionizing radiation can stimulate long-term iron accumulation in lung tissue.

## 4. Discussion and Conclusions

The goal of this study was to investigate the utility of a single-lung-targeted irradiation approach to induce long-term iron accumulation in the lungs. Using this model, it was possible to extend the lifespan of mice beyond those traditionally observed using a whole-thoracic irradiation approach (Table 1), as the animals used in this study were able to survive up to 36 weeks with no observable functional deficits. The effect is likely the result of this model leveraging a sub-total irradiation approach, which allows for maintenance of a functional reserve capacity in the unirradiated lung and heart.

Moreover, this model system did allow for the onset of long-term pathological changes, as evidenced by opacities on the CBCT, the quantifiable loss of NALV, and positive fibrosis scoring in the irradiated lung. Furthermore, this study facilitated the observation of long-term iron accumulation and associated iron metabolic changes in the irradiated lungs, which was evident by the positive Prussian blue staining and increased FtH expression. These results are also consistent with canonical iron metabolic regulation, where FtH expression is increased with response to an iron challenge [17].

Due to the developmental nature of this study, these results are highly correlational, and the small sample size is a limitation of this study. Another limitation to this study that needs to be addressed in future experiments is sex. It has been established that sex plays a role in ionizing-radiation-induced injury, where females appear to be more resistant to injury and males more prone to fibrosis due to differential gene expression and signal transduction activation, such as the renin–angiotensin axis [40,41]. However, the biological results of this exploratory study remain intriguing and suggest that iron metabolism may be involved in the onset of RILI, which warrants further consideration in more robust studies.

The development of this model has translational potential as it may allow for the testing of fundamental mechanisms (e.g., iron metabolic changes) or interrogating therapeutic interventions. Radiation therapy is central to the treatment of lung cancer. However, the dose-limiting effects of RILI and its life-threatening impacts make it vital to elucidate the biological mechanisms at play in order to develop novel therapeutics and improve radiation treatment plans. Currently, the underlying role of iron metabolism in the onset and progression of RILI remains unclear. However, it is not unfounded to consider that iron accumulation plays a contributing role, as transbronchial iron chelation has been shown to ameliorate bleomycin-induced pulmonary fibrosis [42]. Therefore, identifying the role of iron metabolism in RILI will address this current knowledge gap in the field and provide a novel mechanistic insight that may be further explored in the context of different radiation fractionation schemes (e.g., SBRT and conventional fractionation), role of sex, and molecular pathways, which are currently underexplored areas of research. Thus, the use of a single-lung-targeted approach may be useful to evaluate long-term pathological changes in the lung following irradiation by facilitating more robust biochemical analysis. This technical approach may be useful for further investigations into underlying biological mechanisms and preclinical approaches to mitigate RILI.

## Figures and Tables

**Table 1:** Comparison of overall survival using targeted irradiation to studies using whole thoracic radiation.

Prescribed Dose (Gy)	Mean Target Dose (Gy)	Study Duration (Weeks)	Survival of Irradiated Animals at End of Study (%)	Median Survival Time of Irradiated (Weeks)	First Author (Year)
25	17.2 ± 1.4	36	60%	Not reached	(This Report)
20	N/A	17	32%	13.4	[28]
20	N/A	21	0%	17.5	[29]
20	N/A	24	0%	17	[30]
20	N/A	26	14%	17	[31]
20	N/A	35	0%	21.0	[32]
20	N/A	40	0%	19.0	[33]
20	N/A	40	0%	19.0	[34]

## Data Availability

Raw data available upon request.
